# Atrioventricular node ablation for refractory atrial fibrillation in patients with hypertrophic cardiomyopathy: a case–control study

**DOI:** 10.1093/europace/euaf208

**Published:** 2025-09-06

**Authors:** Julia G Debertin, William H Swain, Brett C Austin, Jose F de Melo, Samuel J Asirvatham, Yong-Mei Cha, Freddy Del-Carpio Munoz, Abhishek J Deshmukh, Christopher DeSimone, Fatima M Ezzeddine, Paul A Friedman, Jeffrey B Geske, John R Giudicessi, Suraj Kapa, Ammar M Killu, Gurukripa G Kowlgi, Malini Madhavan, Siva K Mulpuru, Thomas M Munger, Duy T Nguyen, Alan M Sugrue, Nicholas Y Tan, Steve R Ommen, Peter A Noseworthy, Konstantinos C Siontis

**Affiliations:** Mayo Clinic Alix School of Medicine, Mayo Clinic Rochester, Rochester, MN, USA; Department of Cardiovascular Medicine, Mayo Clinic Rochester, 200 First Street S.W., Rochester, MN 55905, USA; Department of Cardiovascular Medicine, Mayo Clinic Rochester, 200 First Street S.W., Rochester, MN 55905, USA; Department of Cardiovascular Medicine, Mayo Clinic Rochester, 200 First Street S.W., Rochester, MN 55905, USA; Department of Cardiovascular Medicine, Mayo Clinic Rochester, 200 First Street S.W., Rochester, MN 55905, USA; Department of Cardiovascular Medicine, Mayo Clinic Rochester, 200 First Street S.W., Rochester, MN 55905, USA; Department of Cardiovascular Medicine, Mayo Clinic Rochester, 200 First Street S.W., Rochester, MN 55905, USA; Department of Cardiovascular Medicine, Mayo Clinic Rochester, 200 First Street S.W., Rochester, MN 55905, USA; Department of Cardiovascular Medicine, Mayo Clinic Rochester, 200 First Street S.W., Rochester, MN 55905, USA; Department of Cardiovascular Medicine, Mayo Clinic Rochester, 200 First Street S.W., Rochester, MN 55905, USA; Department of Cardiovascular Medicine, Mayo Clinic Rochester, 200 First Street S.W., Rochester, MN 55905, USA; Department of Cardiovascular Medicine, Mayo Clinic Rochester, 200 First Street S.W., Rochester, MN 55905, USA; Department of Cardiovascular Medicine, Mayo Clinic Rochester, 200 First Street S.W., Rochester, MN 55905, USA; Department of Cardiovascular Medicine, Mayo Clinic Rochester, 200 First Street S.W., Rochester, MN 55905, USA; Department of Cardiovascular Medicine, Mayo Clinic Rochester, 200 First Street S.W., Rochester, MN 55905, USA; Department of Cardiovascular Medicine, Mayo Clinic Rochester, 200 First Street S.W., Rochester, MN 55905, USA; Department of Cardiovascular Medicine, Mayo Clinic Rochester, 200 First Street S.W., Rochester, MN 55905, USA; Department of Cardiovascular Medicine, Mayo Clinic Rochester, 200 First Street S.W., Rochester, MN 55905, USA; Department of Cardiovascular Medicine, Mayo Clinic Rochester, 200 First Street S.W., Rochester, MN 55905, USA; Department of Cardiovascular Medicine, Mayo Clinic Rochester, 200 First Street S.W., Rochester, MN 55905, USA; Department of Cardiovascular Medicine, Mayo Clinic Rochester, 200 First Street S.W., Rochester, MN 55905, USA; Department of Cardiovascular Medicine, Mayo Clinic Rochester, 200 First Street S.W., Rochester, MN 55905, USA; Department of Cardiovascular Medicine, Mayo Clinic Rochester, 200 First Street S.W., Rochester, MN 55905, USA; Department of Cardiovascular Medicine, Mayo Clinic Rochester, 200 First Street S.W., Rochester, MN 55905, USA; Department of Cardiovascular Medicine, Mayo Clinic Rochester, 200 First Street S.W., Rochester, MN 55905, USA

Atrial fibrillation (AF) is a common arrhythmia in hypertrophic cardiomyopathy (HCM).^1,2^ Catheter ablation of AF is frequently utilized in HCM, but early and late AF recurrences are common, and repeat ablations are often necessary.^3^ In some HCM patients with AF, permanent pacemaker implantation and atrioventricular node ablation (AVNA) may be resorted to (pace-and-ablate strategy). There are limited data regarding the outcomes of this approach in HCM. In a retrospective analysis of HCM patients undergoing AVNA, symptom improvement was noted without decline in left ventricular ejection fraction (LVEF).^4^ However, that study did not include a control cohort. The current study aimed to evaluate LV function and clinical outcomes after AVNA in HCM patients and matched non-HCM controls.

We conducted a retrospective case–control study of consecutive HCM patients, with a diagnosis verified by expert HCM clinicians in accordance with current guidelines,^5^ who underwent AVNA for refractory AF at Mayo Clinic, Rochester, MN (2008–2023). Consecutive patients without HCM who underwent AVNA were also identified. HCM and non-HCM patients were matched 1:4 on age (±5 years) and sex. The study was approved by the Mayo Clinic Institutional Review Board.

The primary outcome was LVEF change after AVNA. Left ventricular ejection fraction was quantified with the transthoracic echocardiography (TTE)-based Simpson’s method at the latest TTE prior to AVNA (LVEF_pre_) and the last TTE available after AVNA (LVEF_post_). Secondary outcomes included heart failure (HF)-related hospitalization, sustained ventricular tachycardia or fibrillation, cardiac resynchronization therapy (CRT) upgrade, cerebrovascular event, and all-cause mortality. The change in LVEF after AVNA in patients with and without HCM was assessed with non-parametric paired Wilcoxon signed-rank testing. Predictors of standardized change in LVEF [(LVEF_post_ − LVEF_pre_)/LVEF_pre_] in HCM patients were assessed with regression analysis.

**Figure 1:**
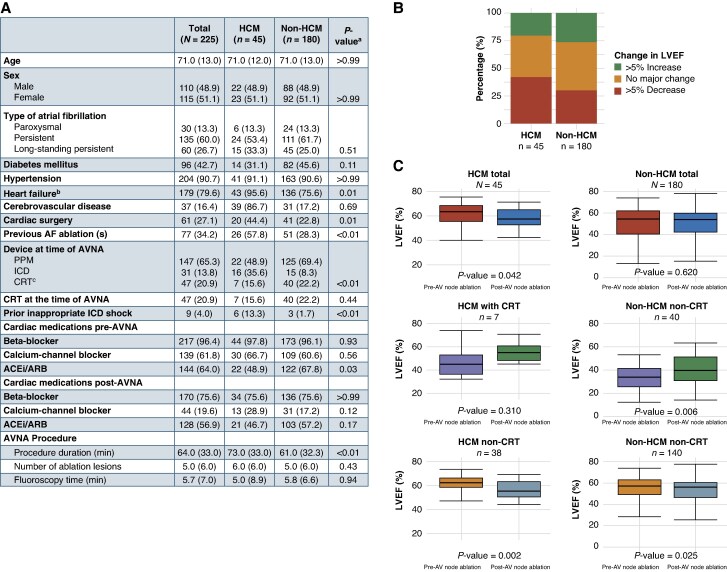
(*A*) Patient characteristics. ^a^*P*-value from χ^2^ test or Wilcoxon signed-rank test. ^b^Includes heart failure with reduced or preserved ejection fraction. ^c^Includes CRT-P and CRT-D. (*B*) Proportions of patients with LVEF change after AV node ablation. The bar graphs depict the proportions of HCM and non-HCM patients with and without significant (>5%) change in LVEF after AV node ablation compared to baseline. Nineteen (42.2%) patients in the HCM group and 54 (30.0%) patients in the non-HCM group (*P* = 0.165) had a decline in LVEF by more than 5%. (*C*) Summarized LVEF changes after AV node ablation in HCM and non-HCM patients according to CRT status. Each box represents the interquartile range (IQR) of LVEF with the dissecting line representing the median LVEF. Error bars are maximum and minimum values or up to ±1.5 * IQR. *P*-values are derived from non-parametric paired Wilcoxon signed-rank test. There was a significant decrease in LVEF after AV node ablation among HCM patients, with a more prominent decrease in the group with non-CRT devices. AVNA, atrioventricular node ablation; HCM, hypertrophic cardiomyopathy; AVN, atrioventricular node; AF, atrial fibrillation; LV, left ventricle; LA, left atrium; PPM, permanent pacemaker; ICD, implantable cardioverter-defibrillator; CRT, cardiac resynchronization therapy.

The cohort consisted of 225 patients (51% female, mean age 70 ± 10.7 years), including 45 patients with HCM who were matched to 180 non-HCM patients (*Figure 1A*). The median interval from baseline TTE to AVNA was 2.4 months, while the median interval from AVNA to follow-up TTE was 35.0 months. The median LVEF_post_ was higher in the HCM compared to the non-HCM group (57.0% vs. 54.0%; *P* < 0.01). Nineteen (42.2%) HCM patients and 54 (30.0%) non-HCM patients had LVEF decline > 5% compared to baseline (*P* = 0.165; *Figure 1B*). For HCM patients with detectable LVOT gradient at baseline (*n* = 11), the pre-AVNA median LVOT gradient was 48.0 (IQR 25.0) mmHg, and the post-AVNA median gradient was 0.0 (IQR 9.5) mmHg (*P* < 0.01).

LVEF_post_ was lower compared to LVEF_pre_ in the HCM patients (median 57% vs. 63%; *P* = 0.04) (*Figure 1C*). The LVEF reduction was most prominent among the 38 HCM patients with non-CRT devices (from median 64 to 57%; *P* = 0.002). The 140 non-HCM patients with non-CRT devices had a small but statistically significant LVEF decline, while those with CRT devices (*n* = 40) had a significant LVEF improvement. Eighteen and 88 patients in the HCM and non-HCM groups died from any cause, with incident mortality rates of 13 and 9.9 per 100 person-years, respectively (*P* = 0.26). There were no significant differences for the other secondary outcomes between the two groups. There was no association between LVEF decline and risk of HF hospitalization in either of the groups.

In the HCM group, beta-blocker therapy was significantly associated with a standardized increase in LVEF [linear coefficient 0.14 (95% CI 0.02, 0.25)]. Having a non-CRT device was significantly associated with a standardized decrease in LVEF [−0.21 (−0.34, −0.07)], as were the pre-AVNA LVEF [−0.01, (−0.0128, −0.004) per 1%] and pre-AVNA septal thickness [−0.02 (−0.03, −0.01) per 1 mm]. Obstructive HCM physiology and sex were not significantly associated with LVEF change.

In this matched case–control analysis of patients with HCM and non-HCM controls undergoing AVNA for refractory AF, there were no significant differences in major clinical outcomes, though HCM patients had greater LVEF reduction post-AVNA, which was driven by the group of HCM patients with non-CRT devices.

This is the first study to evaluate post-AVNA outcomes in HCM compared to non-HCM patients. Atrioventricular node ablation can be effective in the management of refractory AF-related symptoms.^6^ However, the potential adverse effect of chronic RV pacing is an important consideration in patient selection for the pace-and-ablate strategy. Evidence indicates that HCM is associated with a higher degree of left ventricle (LV) dyssynchrony, even with preserved LV systolic function.^7^ A disease-specific predisposition to dyssynchrony may explain the greater decrease in LVEF with chronic pacing.

In a previous study by Butcher *et al.*^4^ including 59 HCM patients who underwent AVNA, the LVEF remained stable even in those without CRT. There are distinct differences between the two studies, the main being the greater degree of LV systolic dysfunction in the Butcher study where the average LVEF in HCM patients was ∼50%, as opposed to 63% in our study. This indicates a high prevalence of patients with end-stage HCM and/or AF-mediated LV systolic dysfunction in that study such that the control of ventricular rates with AVNA may offset the effects of chronic pacing on LV function. Accordingly, a higher baseline LVEF predicted greater LVEF reduction post-AVNA in our study. Further, most HCM patients in the Butcher study had CRT devices at baseline compared to only 16% in our study, which may have mitigated potential trends towards LVEF reduction post-AVNA.

Our results have potential implications for device selection in patients with HCM planned to undergo AVNA. A higher LVEF threshold than the typical 50% for resorting to CRT might be considered in these patients. Similarly, earlier CRT upgrade may be considered before the LVEF drops <50% in the HCM patient. Conduction system pacing (CSP) may be appropriate to consider as a first-line approach in the HCM patient undergoing AVNA.^8^

We acknowledge certain limitations. First, while the AVNAs occurred during a contemporary 15-year period, the rate of CSP was low in accordance with pacing approaches for patients undergoing AVNA in our institution through 2023. Further, this study did not assess functional outcomes including exercise capacity, quality of life, and symptom profiles as these are unreliable to ascertain in retrospective analysis. Finally, due to the sample size, our study was likely underpowered to convincingly rule out differences in major clinical endpoints despite the observed differences in LVEF reduction between the groups.

In conclusion, in this retrospective case–control study, patients with HCM showed similar overall outcome after AVNA compared to patients without HCM. However, in the absence of CRT, AVNA was associated with greater reduction of systolic LV function in HCM patients. Considering the study design, these findings are hypothesis generating, and the role of AVNA in HCM requires randomized evaluation for more definitive guidance.
